# Quantifying met and unmet health needs for HIV, hypertension and diabetes in rural KwaZulu-Natal, South Africa

**DOI:** 10.21203/rs.3.rs-2702048/v1

**Published:** 2023-03-21

**Authors:** Urisha Singh, Stephen Olivier, Diego Cuadros, Alison Castle, Yumna Moosa, Jonathan Alex Edwards, Hae-Young Kim, Mark J. Siedner, Frank Tanser, Emily B. Wong

**Affiliations:** 1 Africa Health Research Institute, KwaZulu-Natal, South Africa; 2 Nelson R Mandela School of Medicine; University of KwaZulu-Natal, Durban, SA; 3 Digital Epidemiology Laboratory, Digital Futures, University of Cincinnati, USA; 4 Division of Infectious Diseases, Massachusetts General Hospital, Boston, MA, USA; 5 Harvard Medical School, Boston, MA, USA; 6 International Institute for Rural Health, University of Lincoln, Lincolnshire, UK; 7 Department of Biostatistics and Bioinformatics, Rollins School of Public Health, Emory University, Atlanta, GA, USA; 8 Department of Biomedical Informatics, Emory University School of Medicine, Emory University, Atlanta, GA, USA; 9 Department of Population Health, New York University Grossman School of Medicine, NY, USA; 10 School of Clinical Medicine, College of Health Sciences, University of KwaZulu-Natal, Durban, South Africa; 11 School of Nursing and Public Health, College of Health Sciences, University of KwaZulu-Natal, Durban, South Africa; 12 Centre for the AIDS Programme of Research in South Africa (CAPRISA), University of KwaZulu-Natal, South Africa; 13 Division of Infectious Diseases, University of Alabama Birmingham, Birmingham, AL, USA

**Keywords:** Multimorbidity, HIV, diabetes, hypertension, health needs, South Africa

## Abstract

**Background:**

The convergence of infectious and non-communicable diseases (NCDs) in South Africa poses a challenge to health systems. Here we establish a framework to quantify met and unmet health needs for individuals living with infectious and NCDs.

**Methods:**

We screened adult residents >15 years of age within the uMkhanyakude district in KwaZulu-Natal, South Africa for HIV, hypertension (HPTN) and diabetes mellitus (DM). For each condition, individuals were defined as having no unmet health needs (absence of condition), met health need (condition that is well controlled), or one or more unmet health needs (including diagnosis, engagement in care, or treatment optimization). We analyzed met and unmet health needs for individual and combined conditions and investigated their geospatial distribution.

**Findings:**

Of 18,041 participants, 9,898 (55%) had at least one chronic condition. 4,942 (50%) of these individuals had at least one unmet health need (18% needed treatment optimization, 13% needed engagement in care, and 19% needed diagnosis). Unmet health needs varied by disease: 93% of people with DM, 58% of people with HPTN and 21% of people with HIV had unmet health needs. Geospatially, met health needs for HIV were widely distributed, unmet health needs had specific sites of concentration whilst the need for diagnosis for all three conditions was co-located.

**Interpretation:**

Whilst people living with HIV are predominantly well-controlled, there is a high burden of unmet health needs for people living with HPTN and DM. Adaptation of HIV models of care to integrate HIV and NCD services is of high priority.

**Funding:**

Fogarty International Center / NIH (R21TW01167, D43TW010543 K24HL166024), Bill and Melinda Gates Foundation, the South African Department of Science and Innovation, South African Medical Research Council and South African Population Research Infrastructure Network (SAPRIN). This research was funded in part, by the Wellcome Trust [Grant number 201433/Z/16/A]. For open access, the author has applied a CC by public copyright license to any Author Accepted Manuscript version arising from this submission.

## Background

Infectious diseases, including HIV and tuberculosis (TB), have dominated the burden of disease in sub-Saharan Africa (SSA) for decades (1). However, like other low- and middle-income countries, regions within SSA are experiencing an epidemiological transition in which prevalence of chronic non-communicable diseases (NCDs) is increasing (2). These NCDs include diabetes mellitus (DM) (3, 4), hypertension (HPTN) and cardiovascular diseases (5, 6), chronic respiratory diseases (7), chronic renal diseases (8), mental and substance use disorders (9, 10) and cancers (11).

Whilst the transition of disease burden has predominantly included shifts from infectious diseases to NCDs globally, numerous studies in South Africa (SA) and some from SSA have reported a convergence of infectious diseases and NCDs (12–17). Managing the convergence of illness is of even greater concern since the advent of COVID-19 because poorly controlled multimorbidity has been associated with a higher risk of severe outcome from COVID-19 (18, 19). Additionally, an increase in ageing among people living with HIV, as a result of the success of antiretroviral therapy (ART), has seen a subsequent increase in NCDs amongst this group resulting in recognition for the need of integrated infectious disease and NCD care and prevention programs in order to avoid a reversal in health gains made through ART (13, 17, 20, 21). The United Nations sustainable development goal number 3, which aims to ensure healthy lives and promote well-being for all at all ages, advocates for the integration of infectious disease and NCD prevention and treatment (22). However, the extent to which health needs of individuals with each of these conditions overlap within individuals and communities, and thus the most efficient and effective approach of designing a health systems response, is not well established.

Here, we used results from a multimorbidity survey conducted in rural SA (12), to assess the multi-disease health needs for individuals and communities in rural KwaZulu-Natal and describe a needs scale which assesses health needs for infectious and NCDs.

## Methods

### Study setting and population

We analysed data from the Vukuzazi study, a cross-sectional health screening survey of individuals aged 15 or older in the uMkhanyakude district of KwaZulu-Natal, South Africa between 2018–2020, which collected information on HIV, DM and HPTN, described previously (12, 23). The Vukuzazi study was embedded within an ongoing Africa Health Research Institute (AHRI) surveillance, the Population intervention Platform (PIP), which conducts multiple household, demographic, and health surveys annually on the surveillance population, and since 2017 has incorporated a clinic surveillance system (ClinicLink) that captures clinic attendance in the 11 primary health facilities in the PIP surveillance area (24). Thus, enabling us to leverage pre-existing demographic, clinic and health data to enrich the Vukuzazi study.

#### Data collected at Vukuzazi

Detailed questionnaires were used to assess individual’s diagnosis and treatment history for each disease. Anthropometric measures and blood pressure were collected according to the WHO STEPS protocol. Blood samples were collected for assessment of glycosylated hemoglobin (HbA1C) and HIV immunoassay testing. Positive HIV immunoassay tests were followed by a reflex HIV-1 RNA viral load assessment (12).

#### Data from PIP and ClinicLink

Data from PIP was taken from the most recent PIP surveillance before Vukuzazi. Socioeconomic status was captured with SES index using principal components analysis. Self-reported measures of perceived overall health, residence status and geolocation of residence was routinely collected as part of the PIP general health and socio-demographic questionnaire. The number of clinic visits individuals made in the year prior to Vukuzazi was obtained through linkage with the ClinicLink system (24). Participants included in the study were geo-located to their respective homesteads using the comprehensive geographic information system (25).

### Definition of the needs score and health states

For this analysis, we defined five health states, based on parallel diagnostic criteria, for each of the three chronic diseases included in this analysis: (i) free of the condition, (ii) diagnosed and optimally treated, (iii) diagnosed and sub-optimally treated, (iv) diagnosed but not engaged in care and (v) undiagnosed but with a positive screening test in Vukuzazi (Table 1).

**Figure 1:**
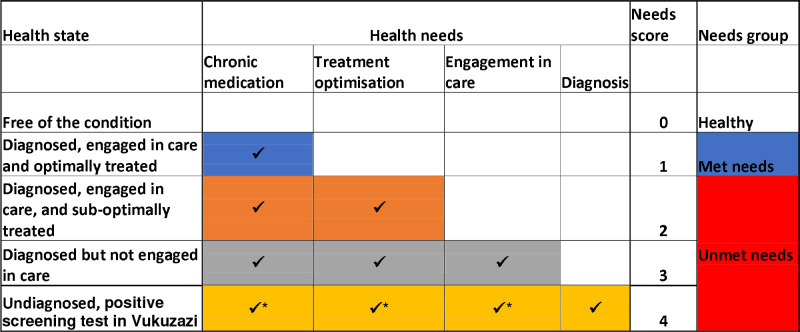
Health states with corresponding health needs, needs scores and needs groups.* dependent on confirmatory diagnostic testing.

The health system needs of each state were captured by a needs score in which the lowest score (zero) represented absence of disease and thus no immediate needs from the health system and the highest score (four) represented individuals who had the highest health needs and required diagnosis, engagement in care, treatment optimization and provision of chronic medication (Figure 1).

Individuals with a needs score of 1, who were diagnosed with the health condition, engaged in care and had their condition optimally controlled on chronic medications were defined as having met health needs. Individuals who had needs score of 2–4, represented those individuals who required one or more health need not currently in place (Figure 1) and were defined as having unmet health needs. Need scores were calculated for individual diseases and for all three diseases combined. In the combined analysis, individuals with more than one disease were assigned the highest needs score for each of their diseases.

### Geospatial analysis

Data visualization analysis of the distribution of health needs for each condition and for all three diseases combined were generated using continuous surface maps of the prevalence distribution of each need. Spatial interpolations were generated with the use of a standard Gaussian kernel interpolation method (with a search radius of 3 km), which has been used and validated in this population for mapping multiple HIV outcomes in the area of study [25]. Maps were created using the ArcGIS Pro software (http://www.esri.com).

### Statistical analysis

We described the prevalence of each need score by disease and for all diseases combined. We then compared the descriptive features of individuals falling within each combined need score. Need scores were compared using Pearson’s Chi-squared test and non-parametric data were analysed using the Kruskal-Wallis rank sum test. Statistical analyses were done in the R statistical software (version 4.2.1).

### Ethical considerations

The Vukuzazi study was approved by the University of KwaZulu-Natal Biomedical Research Ethics Committee and the institutional review board of Mass General Brigham. Written consent was obtained from all participants

## Results

**Figure 2:**
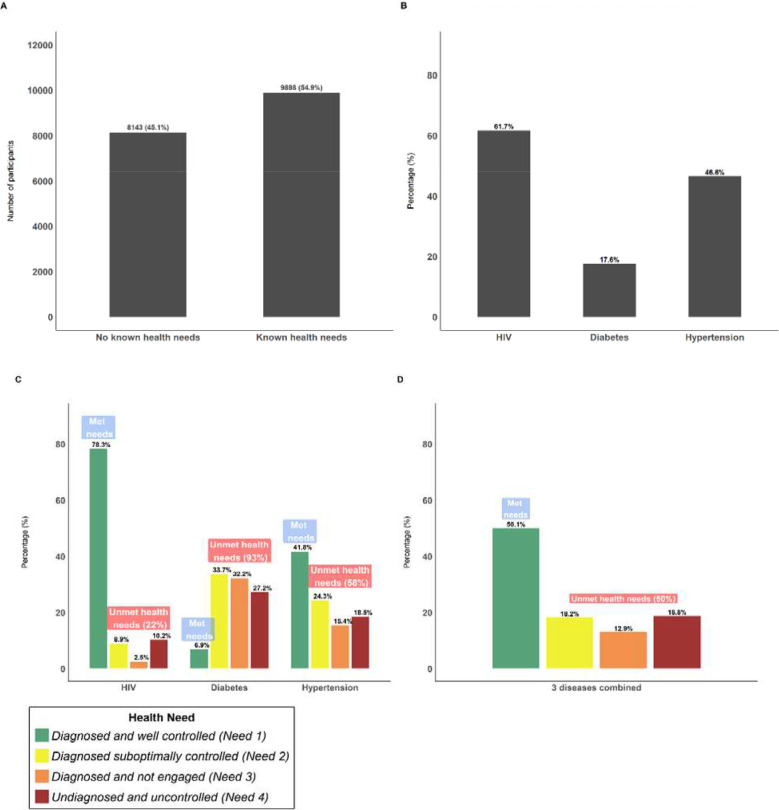
Distribution of health needs for within the Vukuzazi cohort for participants with HIV, diabetes and/or hypertension. (2a) Total number of participants with no health needs identified (n=8143) and those with health needs identified (n=9898) in a cohort of 18041 participants. (2b) Disease distribution amongst individuals with health needs identified (n=9898), 61.7% of these participants had HIV whilst 17.6% and 45.6% of participants with health needs had diabetes or hypertension. (2c) Distribution of met and unmet health needs for individual chronic health states. (2d) Distribution of met and unmet health needs for all three diseases combined (i.e. HIV, diabetes and hypertension).

A total of 18,041 individuals were enrolled in the study, representing 50% of the 36,097 eligible residents of the Demographic surveillance area (DSA) who were >15 years of age. Over half of participants (54.9%, 9,898/18,041) had at least one of the three health conditions measured (Figure 2a). Of those individuals with health conditions, 61.7% had HIV, 46.6% had HPTN and 17.6% had DM (Figure 2b).

Of the participants found to have a chronic health condition, the patterns of met and unmet health needs differed by individual illness (Figure 2c). While 78.3% of HIV-positive participants had their health needs met (diagnosed and utilising chronic medication for optimal disease control), only 6.9% of participants with DM and 41.8% of participants with HPTN had their health needs fully met (Figure 2c). Unmet health needs for individuals with HIV was predominantly driven by the need for treatment optimisation and diagnosis (need score 4), with few participants (2.5%) requiring engagement in care (need score 3). By contrast, for HPTN and DM, all three unmet needs, including engagement in care, contributed significantly to the high levels of unmet health needs. Although 66% and 40% of participants with DM and HPTN were aware of their diagnosis, respectively, they either received suboptimal treatment (need score 2) or were not engaged in care (need score 3, Figure 2c).

When we assessed the health needs of the population for all three disease conditions combined, we found that of the 55% of participants who had at least one of the three health conditions, half had their health needs met and half had at least one unmet health need (Figure 2d). Among those participants with unmet health needs, 18.2% were diagnosed and on treatment that required optimization (need score 2), 12.9% were diagnosed but not engaged in care (need score 3) and 18.8% were undiagnosed and were therefore in need of further diagnostic testing, engagement in care, optimisation of treatment, provision of chronic medication and routine monitoring (need score 4) (Figure 2d).

Distribution of sex, age, BMI, perceived general health state, number of clinic visits in the last year, distance to nearest clinic, residence location and socio-economic status differed between people with no health needs and people with different health needs scores (Table 2). Two thirds of participants were female (68%, 12,229/18,041). With regards to age, results showed a distribution of health needs that varied between age categories. For example, 25–44-year-olds represented the plurality of the people with well-controlled chronic disease (needs score 1, 47%) and undiagnosed chronic disease (needs score 4, 37%) whereas 45–64-year-olds represented the plurality of people with suboptimally controlled chronic disease (needs score 2, 42%) and chronic disease that was diagnosed but not treated (needs score 3, 43%).

Nearly 56% of the cohort were overweight or obese (i.e., BMI > 25kg/m2). These individuals were underrepresented among those without health needs (44%) and over-represented among those with each of the health needs: needs score 1 (61%), needs score 2 (72%), needs score 3 (77%) and needs score 4 (68%) (Table 2).

Despite having unmet health needs, participants with undiagnosed and uncontrolled illnesses had an overall perception of good or very good health. A majority of participants (87%) who were undiagnosed and had uncontrolled illness (need score 4) reported during the PIP survey that they perceived their health as good (55%) or very good (32%) (Table 2). Similarly, 72% of participants who required optimization of treatment (needs score 2) and 75% of participants who required engagement in care (needs score 3), and thus were collectively deemed to be poorly controlled, reported their perceived health status as good or very good.

Many individuals with unmet health needs had visited a clinic in the year prior to engaging in the Vukuzazi study (Table 2). Overall, 42% of participants who were undiagnosed and uncontrolled (need score 4), 57% of participants who required engagement in care (need score 3) and 72% of participants who required optimisation of treatment (need score 2) visited a clinic in the past year, with most participants with unmet health needs having ≥2 visits (Table 2).

Individuals who resided in rural areas were over-represented among people who had diagnosed chronic illness but were not engaged in care (needs score 3); they comprised 86% of people in this needs group compared to 60%, 58% and 49% of needs score groups 1, 2 and 4 respectively (Table 2, p<0.001). People with the furthest distance to the nearest clinic were similarly over-represented among people who were diagnosed but not engaged in care (needs score 3): the median distance to the nearest clinic for these individuals was 3.29km compared to 2.46km, 2.53km and 2.27km for needs score 1, 2 and 4 respectively.

**Figure 3:**
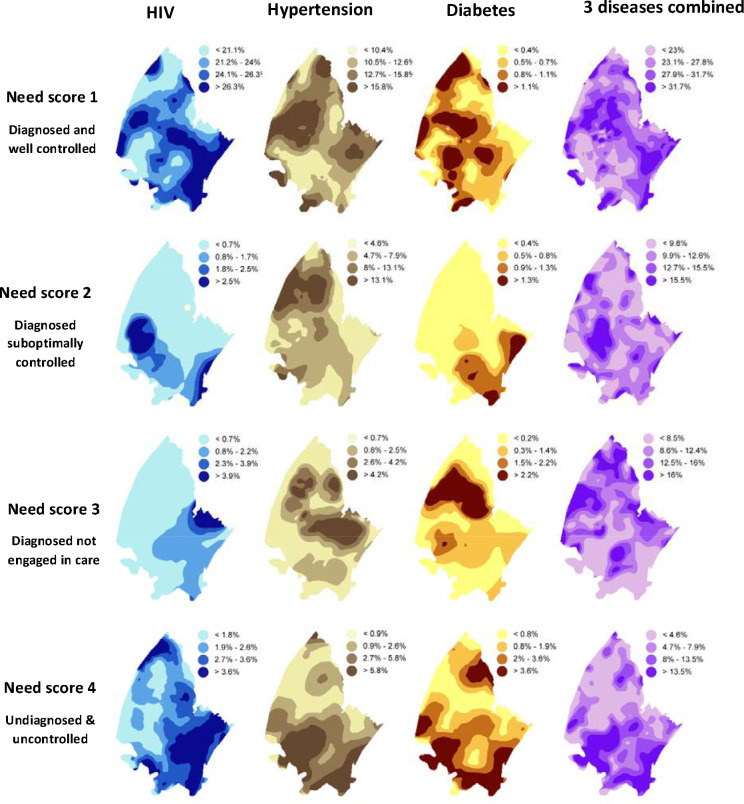
Geospatial distribution of health needs for HIV, hypertension and diabetes individually and for all three chronic conditions combined. Need score 1 reprsenets participants that are diagnosed and well controlled with a need for chronic medicaiton provision. Need score 2 represents participants who are diagnosed and suboptimally controlled with a need for treatment optomisation. Need score 3 represents participants who are diagnosed and not engaged in care with a need for engagement in care. Need score 4 represents participatns who are undiagnosed and uncontrolled with a need for diagnosis, engagement in care, optomisation of treatment and provision of chronic medication.

Having previously observed the lack of geospatial overlap for prevalence of HIV, DM and HPTN within this DSA (12), we sought to assess the geospatial distribution of health needs for these conditions in this area (Figure 3). Needs score 1 was widely distributed throughout the DSA indicating that the need for chronic medication is present across the entire DSA for all three conditions. In contrast needs score 2 and 3 (the need for treatment optimisation and engagement in care respectively), were specifically concentrated in the more rural areas of the demographic surveillance area for all three conditions. Specifically, the need for optimisation of treatment for HPTN and the need for engagement in care for HPTN and DM was concentrated in the northern portion of the surveillance area and the need for optimisation of treatment for DM was higher in the south-eastern portion of the surveillance area. Needs scores 2 and 3 had low density in the Southern-eastern corner of the DSA, the most densely populated region in the surveillance area, while needs score 4 showed overlap for all three conditions within this region indicating a possible target area for diagnostic interventions (Figure 3).

## Discussion

Leveraging data from a large community-based multi-disease survey in rural KwaZulu-Natal, we introduce and implement a health needs framework to conceptualize met and unmet health needs of communities impacted by the overlapping infectious and NCD epidemics in SA. The framework allows determination of comparable needs across chronic disease and promotes comparison by sociodemographic and other health determinants. In our cohort in rural SA, we found that approximately half of people living with chronic illness in this community have unmet health needs. Use of this health needs framework also allows geographic visualizations that illustrate co-localization of individuals with undiagnosed infectious and NCDs. Geospatial data visualization by health needs also illustrates that analyzing populations by their health needs provides useful disaggregation that is obscured when people with a given condition are analyzed in a group without regard to their health needs. Importantly, our framework demonstrates that analysing chronic illness separately and implementing public health approaches in silos misses the opportunity for integration of communicable and NCD chronic care. Consideration should be given to health systems designed to be agnostic to conditions and to people suffering from multiple chronic diseases.

We found that over half of the individuals that engaged in community-based health screening had at least one health need for the diagnosis or management of HIV, DM or HPTN, but that the met or unmet status of these needs differed markedly between HIV and NCDs. Over 70% of the participants with HIV, who were widely distributed throughout the geospatial area, were well controlled on ART. This demonstrates the successful public health response to HIV in its ability to diagnose, optimally treat and monitor patients with a chronic infection across a large rural area. However, it also highlights the stark contrast between HIV and NCD responses: 93% of people who screened positive for diabetes and 58% of people who screened positive for hypertension have unmet health needs in this same community. The lack of NCD disease control is in line with those reported in other studies in the region (26–28). For example, the South African National Health and Nutrition Examination Survey (SANHANES), which considered prevalence of unmet health needs in SA, estimated that 91.5% of hypertensive patients and 80.6% of diabetic patients had an unmet health (26, 27). Smaller studies in the province of Mpumalanga also reported high prevalence of uncontrolled HPTN (between 54.2–56.8%) (28). While these studies assessed health needs of people with HPTN and DM, our study provided a framework for the assessment of these health needs simultaneously with HIV. This revealed a notable discrepancy between the health system’s ability to meet the health needs of people with communicable and NCDs. Our results highlight the massive need for improved NCD care in rural SA, with health systems currently geared to reach a wide target population for HIV, creative adaptation of existing health programs and frameworks could be successful in treating multiple chronic illnesses concurrently.

Unmet health needs also varied by disease and geospatial location in this community. For HIV, most participants with unmet health needs required diagnosis (10.2%) or optimisation of treatment (8.9%). Very few participants (2.5%) required engagement in care, despite a known diagnosis. These data indicate that individuals who have been diagnosed with HIV have mostly been engaged in care and are receiving optimal ART. Conversely, for the NCDs, 65.9% of people who knew they had DM and 39.7% of people who knew they had HPTN required either engagement in care (33.2% and 15.4% for DM and HPTN respectively) or optimisation of treatment (33.7% and 24.3% for DM and HPTN respectively). These differences could partially reflect difficulties in accessing care, since individuals requiring engagement in care tended to live furthest from clinic (3.29km) and were more likely to live in in a rural setting (86%) compared to those with other need scores. In contrast the need for treatment optimisation (need score 2) was strongly associated with increasing age and BMI. Individuals with this health need were predominantly >45 years of age (75%) and were typically overweight (25%) or obese (47%). The association between increased BMI and suboptimal treatment of HPTN, DM or other chronic illnesses has been reported in other studies in which links between obesity, treatment resistant hypertension and altered pharmacological activity of drugs have been reported with use of multiple agents suggested (29). Collectively these data support institution of decentralised patient-centred treatment programs which consider patient parameters like barriers to healthcare access, BMI and age when providing treatment for NCDs.

We found that the need for diagnosis (need score 4) was greater for individuals with DM (27,2%) and HPTN (18.5%) than HIV (10.2%). Individuals with this health need for all three conditions were concentrated in the southern portion of the surveillance area, the most densely populated region in this study. Collectively, this data shows a need to improve access to testing for NCDs. It also shows an opportunity for targeted integrated interventions for NCDs and HIV in this community. There is a possibility that healthcare facilities may have missed opportunities to address the health needs of patients with a diagnosis requiring treatment optimisation (needs score 2) or engagement in care (need score 3) or even undiagnosed patients requiring a diagnosis (need score 4) because the majority of these participants had visited a clinic in the area two or more times in the year before engaging in the Vukuzazi study but still had unmet health needs at the time of the survey also showing the need for improved integrated healthcare.

Our study has several limitations. Firstly, only three chronic disease conditions were considered in this study. Nonetheless, the proposed framework offers flexibility and can be extended to other conditions. Secondly, Vukuzazi only enrolled half of the eligible population which may have biased description of health needs and their associations in directions hard to anticipate based on known differences between the sampled and unsampled population (12). We acknowledge that people who screened positive for DM and HPTN required confirmatory testing prior to confirmation of diagnosis, and that this testing could rule out disease requiring immediate treatment; thus, we may have overestimated the burden of undiagnosed disease (30). Lastly, we acknowledge that it is an oversimplification to ascribe “no health needs” to people who screen negative for disease because it neglects the need for interventions targeting disease prevention, which may be critical for optimal community health.

In summary, we have introduced a needs framework that allows for interrogation of health needs for multiple diseases concurrently despite their individualised prevention, treatment and diagnostic parameters. This novel framework provides a way to conceptualize and measure individual and community health needs for people living in communities with high rates of infectious and NCDs. Applying this framework shows that half of the people living with HIV, DM or HPTN in a South African community have unmet health needs, that the unmet needs are particularly high in people living with NCDs. Furthermore, the granularity of this framework identifies unanticipated geospatial patterns of health need distribution that may inform strategies for improving rural health. New approaches to addressing these unmet health needs are urgently required and we suggest that applying a health needs framework may yield novel insights and guide the design of integrated, decentralized and patient-centered programs for the management of infectious and NCDs.

## Figures and Tables

**Table 1.** Health state definitions for HIV, diabetes mellitus (DM) and hypertension (HPTN).

Health State	HIV	Diabetes Mellitus (DM)	Hypertension (HPTN)

Free of the condition	Immunoassay negative	No previous diagnosis of DM and HBA1c ≤6.5	No previous diagnosis of HPTN, BP<140/<90mmHg
Diagnosed, engaged in care, and optimally treated	Known diagnosis of HIV, on treatment, HIV VL < 40	Known diagnosis of DM, on treatment, HABA1c ≤6.5	Known diagnosis of HPTN, on treatment, BP ≤140/≤90mmHg
Diagnosed, engaged in care, and sub-optimally treated	Known diagnosis of HIV, on treatment, HIV VL > 40	Known diagnosis of DM, on treatment, HBA1c > 6.5	Known diagnosis of HPTN, on treatment, BP ≥140/≥90mmHg
Diagnosed, not engaged in care	Known diagnosis of HIV, not on treatment AND HIV VL > 40	Known diagnosis of DM, not on treatment AND HBA1c>6.5	Known diagnosis of HPTN, not on treatment AND BP≥140/≥90mmHg
Undiagnosed, positive screening test in Vukuzazi	No previous diagnosis of HIV, immunoassay positive, HIV VL>40	No previous diagnosis of DM, HBA1c≥6.5	No previous diagnosis of HPTN, BP≥140/≥90mmHg

**Table 2:** Demographic and socio-economic data disaggregated by health needs.

Characteristic	Overall, N = 18,041^1^	Negative, N = 8,143^1^	Diagnosed and well controlled, N = 4,956^1^	Diagnosed suboptimally controlled, N = 1,802^1^	Diagnosed and not engaged, N = 1,282^1^	Undiagnosed and uncontrolled, N = 1,858^1^	p-value^2^
**Sex (%)**							<0.001
**Male**	5,812 (32%)	3,507 (43%)	973 (20%)	391 (22%)	345 (27%)	596 (32%)	
**Female**	12,229 (68%)	4,636 (57%)	3,983 (80%)	1,411 (78%)	937 (73%)	1,262 (68%)	
**Age**							<0.001
**15–24**	4,962 (28%)	4,152 (51%)	375 (8%)	82 (5%)	59 (5%)	294 (16%)	
**25–44**	6,008 (33%)	2,336 (29%)	2,328 (47%)	367 (20%)	284 (22%)	693 (37%)	
**45–64**	4,595 (25%)	1,104 (14%)	1,626 (33%)	751 (42%)	550 (43%)	564 (30%)	
**65+**	2,476 (14%)	551 (7%)	627 (13%)	602 (33%)	389 (30%)	307 (17%)	
**BMI categories**							<0.001
**Normal (BMI: 18.5–24)**	7,053 (40%)	4,058 (50%)	1,726 (35%)	428 (24%)	265 (21%)	576 (31%)	
**Underweight (BMI <18.5)**	857 (5%)	528 (7%)	195 (4%)	47 (3%)	33 (3%)	54 (3%)	
**Overweight (BMI 25–30)**	4,048 (23%)	1,660 (21%)	1,236 (25%)	448 (25%)	297 (24%)	407 (22%)	
**Obese (BMI >30)**	5,884 (33%)	1,835 (23%)	1,752 (36%)	835 (47%)	662 (53%)	800 (44%)	
**Perceived General Health (last DSS survey)**							<0.001
**Poor to Fair**	2,192 (14%)	494 (7%)	712 (16%)	478 (28%)	293 (24%)	215 (13%)	
**Good**	8,758 (55%)	3,591 (53%)	2,694 (59%)	908 (54%)	657 (54%)	908 (55%)	
**Very good**	4,962 (31%)	2,695 (40%)	1,172 (26%)	299 (18%)	260 (21%)	536 (32%)	
**Any clinic visits past year (ClinicLink)**	9,561 (53%)	2,925 (36%)	3,824 (77%)	1,302 (72%)	725 (57%)	785 (42%)	<0.001
**Number of clinic visits in past year (ClinicLink)**							<0.001
**1**	2,068 (22%)	1,242 (42%)	301 (8%)	151 (12%)	152 (21%)	222 (28%)	
**2–4**	3,084 (32%)	1,105 (38%)	1,153 (30%)	324 (25%)	230 (32%)	272 (35%)	
**5+**	4,409 (46%)	578 (20%)	2,370 (62%)	827 (64%)	343 (47%)	291 (37%)	
**Distance to nearest clinic (KM)**	2.63 (1.52, 4.07)	2.75 (1.62, 4.22)	2.46 (1.47, 3.85)	2.53 (1.42, 4.01)	3.29 (2.08, 4.45)	2.27 (1.34, 3.61)	<0.001
**Smoking status**							<0.001
**Never smoker**	16,573 (92%)	7,383 (91%)	4,622 (93%)	1,692 (94%)	1,168 (91%)	1,708 (92%)	
**Ex-smoker**	150 (1%)	58 (1%)	46 (1%)	19 (1%)	16 (1%)	11 (1%)	
**Current smoker**	1,301 (7%)	685 (8%)	288 (6%)	91 (5%)	98 (8%)	139 (7%)	
**Drinking status**							<0.001
**Never drinker**	15,752 (87%)	7,009 (86%)	4,409 (89%)	1,627 (90%)	1,125 (88%)	1,582 (85%)	
**No drinking in last 12 months**	306 (2%)	154 (2%)	73 (1%)	28 (2%)	23 (2%)	28 (2%)	
**Drinking in past 12 months**	1,966 (11%)	963 (12%)	474 (10%)	147 (8%)	134 (10%)	248 (13%)	
**Household size**							<0.001
**Small-Medium household (1–5)**	12,662 (70%)	5,355 (66%)	3,668 (74%)	1,360 (75%)	920 (72%)	1,359 (73%)	
**Large household (5<)**	5,379 (30%)	2,788 (34%)	1,288 (26%)	442 (25%)	362 (28%)	499 (27%)	
**Residence location**							<0.001
**Rural**	11,436 (64%)	5,430 (67%)	2,951 (60%)	1,049 (58%)	1,104 (86%)	902 (49%)	
**Peri-Urban**	5,599 (31%)	2,342 (29%)	1,672 (34%)	644 (36%)	160 (12%)	781 (42%)	
**Urban**	950 (5%)	347 (4%)	317 (6%)	102 (6%)	16 (1%)	168 (9%)	
**Socioeconomic status**							<0.001
**Low**	6,457 (37%)	2,920 (37%)	1,868 (39%)	626 (36%)	465 (37%)	578 (32%)	
**Middle**	6,043 (35%)	2,762 (35%)	1,652 (35%)	573 (33%)	442 (35%)	614 (34%)	
**High**	4,968 (28%)	2,227 (28%)	1,248 (26%)	545 (31%)	352 (28%)	596 (33%)	

1 n (%); Median (IQR)

2 Pearson’s Chi-squared test; Kruskal-Wallis rank sum test
